# 2,8‑Diphenylbenzo[1,2‑*b*:4,5‑*b*′]bis[*b*]benzothiophene: A New Thienoacene Derivative for Potential Organic Electronic Applications

**DOI:** 10.1021/acsomega.6c04431

**Published:** 2026-07-13

**Authors:** Aneta Rzewnicka, Remigiusz Żurawiński, Jerzy Krysiak, Tomasz Makowski

**Affiliations:** † Division of Organic Chemistry, Centre of Molecular and Macromolecular Studies, 86897 Polish Academy of Sciences, Sienkiewicza 112, 90-363 Lodz, Poland; ‡ Department of Polymeric Nano-Materials, Centre of Molecular and Macromolecular Studies, Polish Academy of Sciences, Sienkiewicza 112, 90-363 Lodz, Poland

## Abstract

This work reports the synthesis and comprehensive characterization of 2,8-diphenylbenzo­[1,2-*b*:4,5-*b*′]­bis­[*b*]­benzothiophene (**diPh-BBBT**), a novel thienoacene derivative designed for organic electronic applications. The compound was synthesized from 1,4-dibromo-2,5-bis­(methylsulfinyl)­benzene via Suzuki–Miyaura cross–coupling reaction, followed by double intramolecular cyclization and demethylation. **diPh-BBBT** demonstrates high thermal stability, pronounced crystallinity, and enhanced resistance to oxidative degradation. In comparison to the benchmark semiconductor 2,7-diphenylbenzothieno­[3,2-*b*]­benzothiophene (**diPh-BTBT**), **diPh-BBBT** exhibits a lower hole reorganization energy (λ_h_ = 0.25 eV), which indicates enhanced hole-transport properties. Charge-transport simulations based on a simplified packing model consistent with XRD data for a mechanically oriented film predict hole mobilities of up to approximately 0.5 cm^2^ V^–1^ s^–1^.

## Graphical abstract

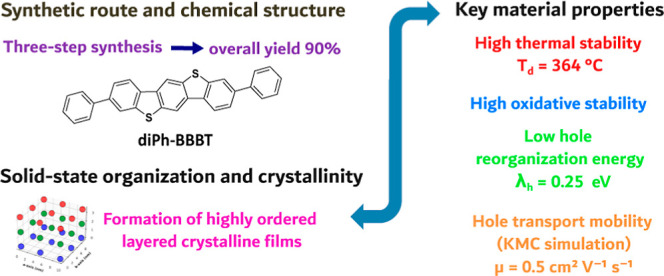

## Introduction

Organic materials with strongly conjugated π–systems have formed the foundation of the rapidly developing field of organic electronics for over two decades.^1,2^ Among these materials, linear π–extended acenes, particularly pentacene and its derivatives, hold a distinct position as efficient charge-transport materials.^3–8^ For higher-order acenes (e.g., hexacene and heptacene), higher mobility is expected; however, their synthesis and material applications are limited due to low chemical stability.^9–11^ To mitigate the instability of large acenes, heteroaromatic units such as thiophene are incorporated into the acene framework in place of selected benzene rings, resulting in more stable thienoacenes. The introduction of sulfur atoms into the structure of acenes lowers the HOMO energy level and increases the band gap, thereby enhancing resistance to oxidation, improving ambient stability, and providing greater protection against photochemical degradation compared with many linear acenes.^12^ Additionally, thienoacenes feature extended π-electronic systems along their backbones and exhibit relatively strong intermolecular π–π interactions, which promote the formation of highly ordered layered crystalline thin films, further facilitating efficient charge transport. These characteristics make thienoacene-based compounds, such as benzothieno­[3,2-*b*]­benzothiophene (**BTBT**),^13–23^ dinaphtho­[2,3-*b*:2′,3′-*f*]­thieno­[3,2-*b*]­thiophene (**DNTT**),^24–28^ benzo­[1,2-*b*:4,5-*b*′]­bis­[*b*]­benzothiophene (**BBBT**),^29–31^ dinaphtho­[2,3-*d*:2′,3′-*d*′]­benzo­[1,2-*b*:4,5-*b*′]­dithiophene (**DNBDT**)^32–34^ the subject of ongoing research interest, as they represent key materials for applications in organic field-effect transistors (OFET), organic light-emitting diodes (OLED), and organic photovoltaics (OPV) (Figure 1).

**Figure 1:**
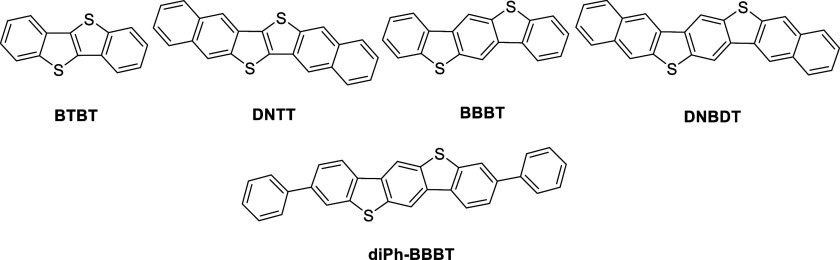
Chemical structures of representative thienoacene cores and **diPh-BBBT**.

The careful design of substituents on these core structures significantly impacts their performance in electronic devices. For instance, **BTBT** derivatives have exhibited charge mobilities reaching 43 cm^2^ V^–1^ s^–1^ ^21^ under ambient conditions due to their favorable intermolecular packing arrangements, which typically adopt either herringbone or π-stacked configurations depending on the nature and position of the substituents. Similarly, **DNTT** derivatives, characterized by an extended π-conjugated core, exhibit excellent performance in field-effect devices, with charge-carrier mobilities of up to 7.5 cm^2^ V^–1^ s^–1^.^25^ In addition, **DNBDT** derivatives have enabled air-stable p-channel OFETs with a maximum hole mobility of 16 cm^2^ V^–1^ s^–1^.^33^

Recently, the OFET properties of **BBBT** and its derivatives have also been investigated.^29–31^ It has been demonstrated that strategic substitution patterns can promote the formation of layered crystal structures, which are critical for high charge mobility. In particular, symmetric **diC10-BBBT** and asymmetric **Ph-BBBT-C10** adopt layered π-stack (LπS) and layered herringbone (LHB) packing motifs, respectively, enabling the formation of uniform (ultra)­thin films with hole mobilities of around 1 cm^2^ V^–1^ s^–1^—a significant advance for this class of materials. These examples illustrate how substituents modulate crystal packing, shifting from the nonlayered motif of unsubstituted **BBBT** to transport-favorable layered arrangements.^31,35^ Building on this design principle, we introduce 2,8-diphenylbenzo­[1,2-*b*:4,5-*b*′]­bis­[*b*]­benzothiophene (**diPh-BBBT**). Compared to the classic **BTBT** system, the **BBBT** core incorporates an additional fused benzene ring, resulting in an inherently more extended π-conjugated framework. Furthermore, the introduction of symmetric phenyl substituents on this expanded core enhances intermolecular interactions and structural order while preserving thermal robustness.

Herein, we report the synthesis, theoretical calculations, and comprehensive investigation of its optical, thermal, and structural properties, identifying **diPh-BBBT** as a promising candidate for organic electronic applications.

The compound was characterized using a range of complementary analytical techniques. Theoretical calculations, utilizing density functional theory (DFT) and time-dependent density functional theory (TD-DFT), provided insights into its electronic properties, including HOMO–LUMO energies, ionization potential, and reorganization energy. Optical properties were investigated through absorption and emission measurements in solution (dichloromethane and dimethyl sulfoxide) and in the solid state. Thermal stability was determined by thermogravimetric analysis (TGA) and differential scanning calorimetry (DSC). Structural organization was analyzed using X-ray diffraction (XRD) and polarized optical microscopy (POM) of mechanically oriented films. Additional morphological characterization was performed using atomic force microscopy (AFM) to evaluate surface features and nanoscale crystalline organization. The charge-carrier mobility was evaluated using the Marcus charge-hopping rate formalism. This comprehensive, multitechnique methodology allows for a thorough assessment of **diPh-BBBT** properties relevant to its potential performance in organic electronic devices.

## Results and Discussion

### Synthesis

The target compound, **diPh-BBBT**, was synthesized using 1,4-dibromo-2,5-bis­(methylsulfinyl)­benzene (**1**) as the key starting material, which was prepared following a previously reported procedure.^36^ The Suzuki–Miyaura cross–coupling reaction between compound **1** and the corresponding biphenyl boronic ester **2** yielded the key intermediate **3** as a mixture of diastereomers in 77% yield. This step proceeded smoothly under standard palladium-catalyzed conditions, demonstrating the compatibility of both coupling partners and the robustness of the reaction protocol.^30^ The synthesis was completed through a two-step transformation. First, intermediate **3** underwent a double intramolecular cyclization in the presence of an excess of CF_3_SO_3_H, resulting in the quantitative formation of the corresponding sulfonium salt. The final step involved the demethylation of the obtained sulfonium salt, which was performed by heating it in pyridine.

This two-step sequence, based on sulfonium salt formation follow by demethylation, is a well-establihed strategy for constructing fused π-conjugated frameworks.^30,37,38^ It proceeded efficiently, affording the target compound **diPh-BBBT** in an excellent yield of 92% (Scheme 1).

**Scheme 1:**
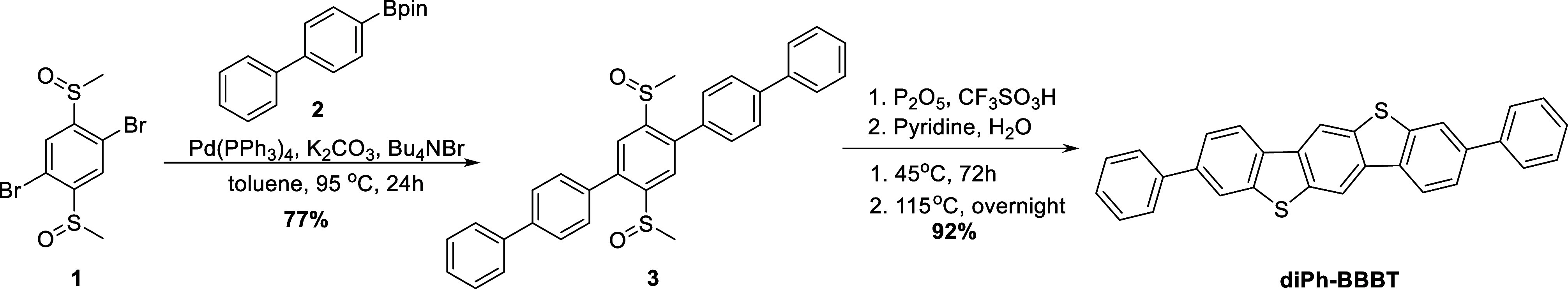
Synthesis of **diPh-BBBT**

### Optical and Thermal Properties

**diPh-BBBT** exhibits maximum absorption and emission in DCM at 344 and 393 nm (with a right shoulder), respectively (Figure 2a). The compound displays a negligible solvatochromic effect in both absorption and emission spectra. Switching the solvent from dichloromethane (DCM) to much more polar dimethyl sulfoxide (DMSO) induces only a minimal bathochromic shift of the corresponding bands by approximately 4 nm (Figure 2b). The solid-state absorption and emission spectra of **diPh-BBBT** exhibit more pronounced shifts in the positions of their maximum absorption and emission bands. Relative to the spectra recorded in DCM, the absorption maximum is red-shifted by 10 nm, while the emission maximum shows a substantially larger red shift of 46 nm (Figure 2c).

**Figure 2:**
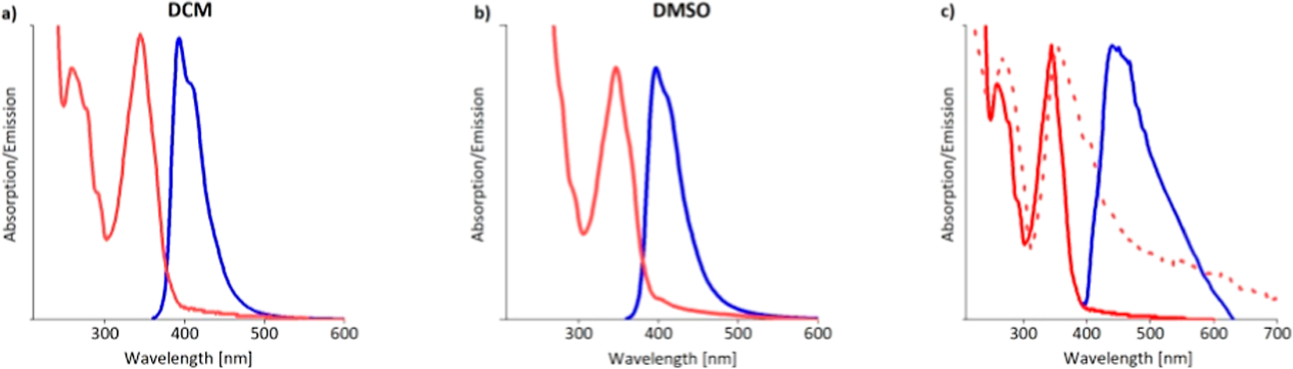
Optical and thermal properties of **diPh-BBBT**, (a) Normalized Ultraviolet–Visible (UV–Vis) (red solid line) and photoluminescence (PL) spectra (blue solid line) in DCM; (b) Normalized UV–Vis spectra (red solid line) and PL spectra (blue solid line) in DMSO; (c) Normalized UV–Vis spectra in DCM (red solid line), UV–Vis (red dotted line) and PL spectra (blue solid line) in solid.

TGA analysis of **diPh-BBBT** revealed high thermal stability, with a 5% weight-loss decomposition temperature (*T*_d_) of approximately 360 °C (Figure S1, Supporting Information), indicating that **diPh-BBBT** can withstand processing conditions typically required for device fabrication, including thermal annealing steps commonly used to optimize thin-film morphology. The gradual weight loss above 200 °C and more significant decomposition above 350 °C suggest a multistep degradation mechanism, possibly involving loss of phenyl groups or ring fragmentation. The thermal stability profile is substantially better than many solution-processable organic semiconductors (e.g., polythiophenes *T*_d_ ≈ 250–300 °C) and comparable to vacuum-deposited small molecules such as pentacene (*T*_d_ ≈ 350 °C) or established **BTBT** derivatives.^39,40^ DSC analysis provided additional insights into the thermal behavior of **diPh-BBBT** (Figure S2, Supporting Information). The DSC curve showed no sharp melting transition, but a broad thermal event is observed at 150–200 °C.

### DFT and TD-DFT Analysis

To assess the electronic characteristics of **diPh-BBBT**, we carried out comparative theoretical analyses using 2,7-diphenyl[1]­benzothieno­[3,2-*b*]­benzothiophene (**diPh-BTBT**) as a benchmark. **diPh-BTBT** is well established in the literature as a *p*-type small-molecule semiconductor, and when used as the active layer in OFETs, it has demonstrated hole mobilities up to approximately 2 cm^2^ V^–1^ s^–1^ under ambient conditions.^13^ Using **diPh-BTBT** as reference gives a sound basis for evaluating how structural modifications in **diPh-BBBT** might impact properties such as electronic energy levels, charge injection barriers, and reorganization energies—all of which are central to estimating charge transport performance in electronic or optoelectronic application. The calculated frontier orbital energies, excitation energies and changes in electron densities are presented in Table 1 and in Figure 3. Expanding the π–conjugation in **diPh-BTBT** by inserting a phenyl ring between the two thiophene units lowers both the HOMO and LUMO energy levels by approximately 0.12 eV, while leaving the overall energy band gap essentially unchanged. This structural modification also slightly decreases the excitation energy of **diPh-BBBT** and consequently shifts its maximum absorption wavelength to longer values, resulting in a red shift of about 5 nm compared with **diPh-BTBT**. For both compounds, the excitation to the first singlet excited state S_1_ is primarily dominated by the HOMO → LUMO transition, although in **diPh-BBBT**, the HOMO-1 → LUMO transition also makes a notable contribution to the excitation of approximately 7.5%.

**Table 1:** Optical and Electronic Properties of **diPh-BTBT** and **diPh-BBBT** Calculated Using the TD-DFT pbe0/6-311+G­(2d,p)//pbe0-D3­(BJ)/6-311G­(2d,p) Level of Theory in DCM (IEFPCM Solvation Model)

compound	λ_abs_ (nm)	*E*_abs_ (eV)	*E*_HOMO_ (eV)	*E*_LUMO_ (eV)	Δ*E*_HOMO–LUMO_ (eV)	main transition
**diPh-BTBT**	361	3.43	–5.94	–1.80	4.14	H → L (98.2%)
**diPh-BBBT**	366	3.39	–6.07	–1.92	4.15	H → L (88.6%); H-1 → L (7.5%)

**Figure 3:**
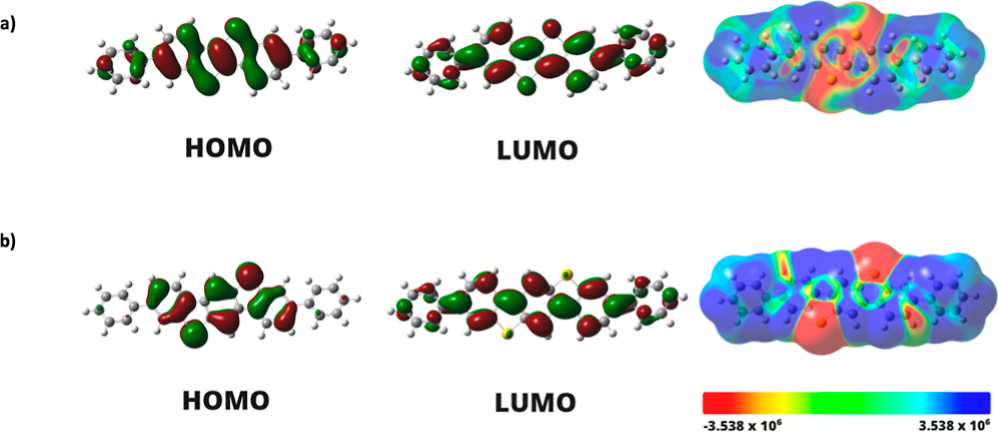
Graphical representation of frontier molecular orbitals (isovalue = 0.024) and electron density changes upon excitation from the ground state to the *S*_1_ excited state for (a) **diPh-BTBT** and (b) **diPh-BBBT**.

The structural differences between **diPh-BTBT** and **diPh-BBBT** have a pronounced impact on the charge distribution within their frontier molecular orbitals. In **diPh-BTBT**, the HOMO is delocalized across the entire π-conjugated framework, indicating efficient conjugation throughout the molecule. In contrast, the HOMO of **diPh-BBBT** is primarily localized on the central five-membered skeleton. Upon excitation to the first excited state, electron transfer from the thienyl sulfur atoms toward the phenyl substituents occurs in both compounds; however, this effect is more pronounced in **diPh-BBBT**.

To gain a deeper understanding of both the ambient stability and charge-transport characteristics of the compounds under investigation, we determined their adiabatic ionization potentials (IPs), electron affinities (EAs), and internal reorganization energies (λ) (Table 2). These electronic parameters offer valuable information about the molecule’s ability to undergo oxidation or reduction. In particular, IP reflects how easily a molecule can lose an electron, whereas EA indicates its tendency to accept one. Together, they play a central role in defining the electronic behavior, redox activity, and overall stability of the materials. Beyond describing intrinsic electronic tendencies, IP and EA also provide a practical framework for estimating the energetic barriers associated with charge injection at interfaces, such as the transfer of holes (from a positive electrode) or electrons (from a negative electrode) into the molecular framework. On the other hand, the reorganization energy (λ) is one of the key parameters for determining the efficiency of charge transport in organic semiconductors and refers to the energy required for the structural and electronic rearrangement that occurs during a charge transfer process. Organic semiconductors with smaller reorganization energy (assuming the other parameters such as intermolecular transfer integrals and molecular arrangement in crystal are similar) generally show a higher charge mobility - due to the lower structural changes between charged and uncharged moieties. The theoretical calculations revealed that **diPh-BBBT** has a higher IP and EA compared to the reference **diPh-BTBT**, implying enhanced resistance toward oxidative degradation. Furthermore, both compounds exhibit lower λ_h_ than λ_e_, which predisposes them to function as p-type semiconductors. Notably, the λ_h_ of **diPh-BBBT** is lower than that of **diPh-BTBT** by 0.06 eV, suggesting more efficient hole-transport characteristic in **diPh-BBBT**.

**Table 2:** Ionization Potentials (IPs), Electron Affinities (EAs), and Reorganization Energies (λ) of **diPh-BTBT** and **diPh-BBBT** Calculated Using the M06-2x-D3/def2-TZVPD Level in the Gas Phase

compound	λ_h_ (eV)	λ_e_ (eV)	λ = λ_h_ + λ_e_	IP (eV)	EA (eV)
**diPh-BTBT**	0.31	0.41	0.72	7.18	0.713
**diPh-BBBT**	0.25	0.32	0.57	7.35	0.843

### Structural Characterization

#### X-ray Diffraction Analysis

XRD analysis of the mechanically oriented **diPh-BBBT** film revealed a well-defined crystalline structure with six distinct diffraction peaks at 2θ = 5.15°, 18.79°, 19.19°, 22.02°, 22.68°, and 26.69°, corresponding to *d*-spacings of 17.16, 4.72, 4.62, 4.04, 3.92, and 3.34 Å, respectively (Table 3 and Figure 4b). The most intense low-angle reflection at 2θ = 5.15° (*d* = 17.16 Å) can be assigned to the layer-to-layer spacing (*d*_001_), strongly supporting a layered packing motif in **diPh-BBBT**. This *d*-spacing is substantially larger than typical π–π stacking distances of about 3.5–4.0 Å and falls within the range expected for phenyl-substituted **BBBT** derivatives, where the substituents increase the interlayer separation. The reflections observed in the intermediate-angle region (18.79°–22.68°) are consistent with in-plane molecular periodicities within the ordered domains, while the high-angle peak at 26.69° (*d* = 3.34 Å) indicates the presence of short intermolecular contacts that may facilitate intermolecular electronic coupling. The presence of closely spaced reflections, such as the 18.79°/19.19° pair, is consistent with mechanically induced texture and preferred molecular orientation in the film, leading to a textured layered organization. Such preferential alignment may be beneficial for directional charge transport along the extended π-conjugated framework. Moreover, the absence of a pronounced diffuse background and the presence of well-resolved diffraction maxima suggest good crystalline order with only a limited amorphous contribution. Tentative indexing suggests the assignments (001), (100), (110), (200), (111), and (300) for the reflections at 5.15°, 18.79°, 19.19°, 22.02°, 22.68°, and 26.69°, respectively. Taken together, these results indicate that mechanically oriented **diPh-BBBT** forms a highly ordered layered structure, which is consistent with its promising charge-transport characteristics.

**Table 3:** XRD Data for **diPh-BBBT** (Mechanically Oriented Film)

reflection	2θ (°)	*d*-spacing (Å)	*d*-spacing (nm)	possible indexing	interpretation
1	5.15	17.16	1.716	(001)	Layer stacking
2	18.79	4.72	0.472	(100)	In-plane aromatic
3	19.19	4.62	0.462	(110)	Textured component
4	22.02	4.04	0.404	(200)	Higher-order
5	22.68	3.92	0.392	(111)	Mixed type
6	26.69	3.34	0.334	(300)	High-angle

**Figure 4:**
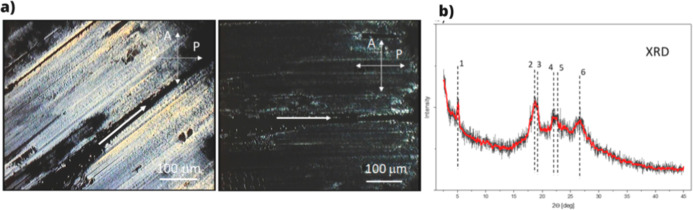
(a) POM image of the mechanically oriented **diPh-BBBT** film before and after rotation by 45°. The white arrow indicates the rubbing direction. (b) XRD pattern of the mechanically oriented film, showing reflections characteristic of layered molecular organization.

#### Polarized Optical Microscopy Analysis

POM imaging of **diPh-BBBT** films revealed a highly ordered, crystalline structure characterized by distinct birefringence and well-defined crystal domains (Figure 4a). Under crossed polarizers, the films displayed interference colors ranging from blue to yellow, indicating varying crystal thicknesses and uniformly oriented crystalline domains within each region. The consistent color within individual domains suggests single-crystalline or highly texturally aligned polycrystalline character within each domain. Mechanical orientation experiments, where **diPh-BBBT** powder was pressed between glass slides under directional rubbing, induced preferential crystalline alignment. These oriented films showed characteristic extinction patterns consistent with a highly anisotropic crystal structure, with the principal optical axes aligned along preferred directions. The extinction angle and birefringence patterns observed in POM indicate a planar molecular arrangement with strong in-plane conjugation, characteristic of organic semiconductors designed for efficient charge transport.

#### AFM Analysis

AFM measurements of **diPh-BBBT** films revealed a well-defined nanoscale surface morphology with ordered crystalline domains (Figure 5). Tapping-mode height images showed distinct features with widths of 60–80 nm, consistent with the large grains observed by POM. Three-dimensional topographic reconstruction revealed crystalline hillocks and well-defined interdomain boundaries. Surface roughness analysis yielded a root-mean-square (RMS) roughness (*R*_q_) of ≈14 nm, indicating a smooth morphology suitable for efficient semiconductor–dielectric interfaces in OFETs.

**Figure 5:**
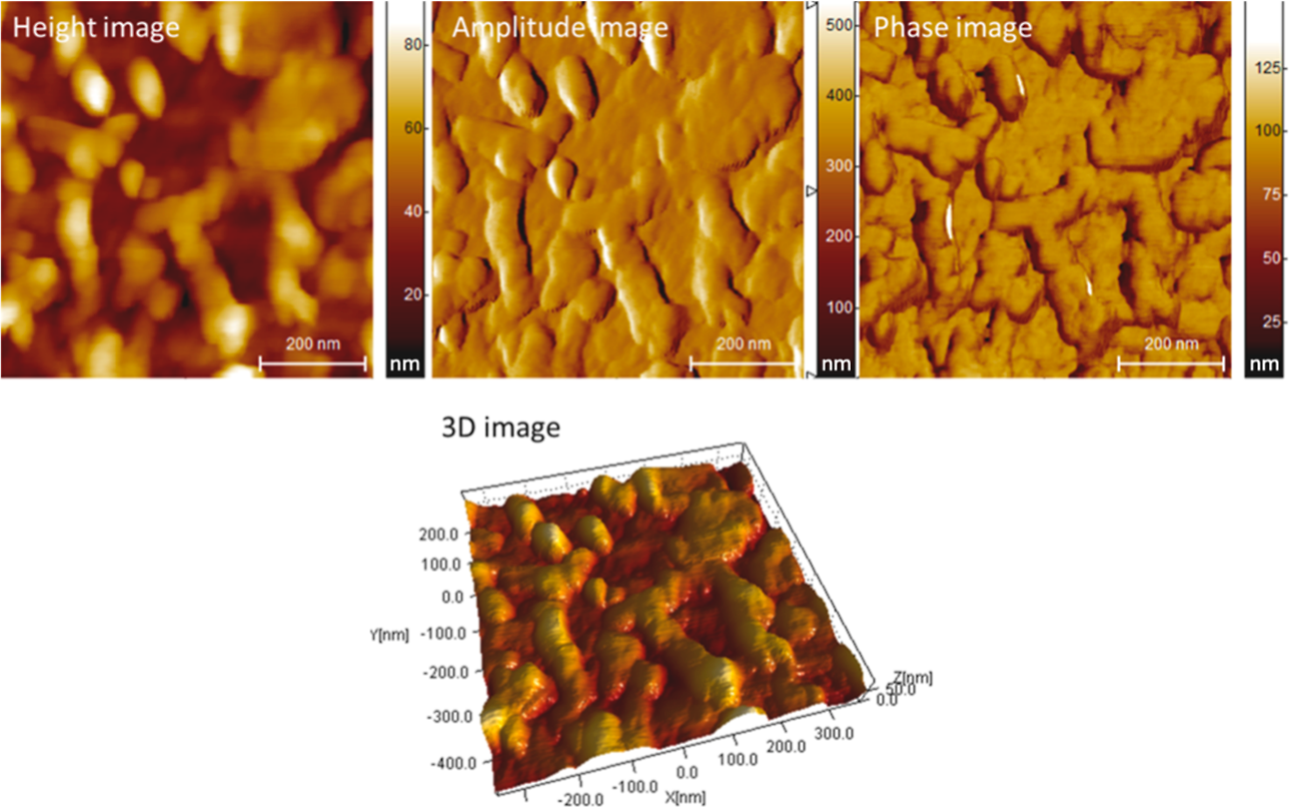
AFM images of **diPh-BBBT** of mechanically oriented layered.

#### Charge Transport Analysis

Charge transport properties of **diPh-BBBT** were assessed indirectly due to its exceptionally low solubility in common organic solvents, which makes the fabrication of high-performance solution-processed OFETs technically challenging. Hole mobility was therefore evaluated using kinetic Monte Carlo (KMC) simulations within the Marcus charge-hopping formalism, based on experimentally derived XRD structural parameters and reorganization energy (λ) from DFT calculations. Simulations were performed at 300 K with disorder σ = 0.04 eV under realistic Si/SiO_2_ (200 nm dielectric thickness)/Vg = −60 V conditions, yielding mobilities of 0.478 cm^2^ V^–1^ s^–1^ (*J* = 69 meV) and 0.253 cm^2^ V^–1^ s^–1^ (*J* = 59 meV). Structural analysis reveals key differences between **diPh-BTBT** and **diPh-BBBT**. **diPh-BTBT** displays an XRD peak at 2θ = 22.21° (*d* ≈ 4.00 Å), characteristic of herringbone packing, and yields a KMC mobility of μ = 0.195 cm^2^ V^–1^ s^–1^ at *J* = 47 meV under the same Si/SiO_2_ (200 nm)/Vg = −60 V conditions, in excellent agreement with literature device data.^13^ In contrast, **diPh-BBBT** exhibits 2θ = 26.69° (*d* ≈ 3.34 Å), indicating closer π–π stacking typical of high-mobility BTBT derivatives. For the same *J* = 47 meV, **diPh-BBBT** gives μ = 0.105 cm^2^ V^–1^ s^–1^, however, literature reports for similar π-stacked systems indicate *J* values up to 80 meV, suggesting substantially higher intrinsic potential (μ > 1 cm^2^ V^–1^ s^–1^).^31^ These values should be regarded as model-based predictions of intrinsic charge-transport behavior derived from KMC simulations, rather than as experimentally measured OFET mobilities. Overall, these results demonstrate that **diPh-BBBT** combines tighter molecular packing with promising charge-transport properties, despite its limited solubility.

## Conclusion

This work demonstrates a high-yielding and operationally robust synthesis of **diPh-BBBT** and provides a comprehensive physicochemical characterization of this new thienoacene-based material for organic electronic applications. Combined experimental and theoretical results show that **diPh-BBBT** exhibits high thermal stability, pronounced crystallinity, and enhanced resistance to oxidative degradation, along with a reduced hole reorganization energy relative to the benchmark semiconductor **diPh-BTBT**. Structural studies further indicate that mechanically oriented **diPh-BBBT** forms highly ordered layered crystalline films, a packing motif consistent with efficient intermolecular electronic coupling and favorable hole-transport characteristics. In line with these observations, kinetic Monte Carlo simulations based on an XRD-informed simplified packing model predict hole mobilities of up to approximately 0.5 cm^2^ V^–1^ s^–1^ under the adopted assumptions. Taken together, these findings identify **diPh-BBBT** as a promising candidate for p-type organic semiconducting materials, although OFET studies will be necessary to verify device-level performance. Although the low solubility of **diPh-BBBT** may hinder straightforward solution-based processing, its high thermal stability makes it a promising candidate for vacuum thermal evaporation; therefore, the fabrication and characterization of vacuum-deposited OFET devices constitute a logical next step to verify its device-level charge-transport efficiency.

## Experimental Section

### General Remarks and Synthetic Procedures

Commercial-grade reagents and solvents were used as received, without further purification. The synthesis of 1,4-dibromo-2,5-bis­(methylsulfinyl)­benzene (**1**) was carried out according to established literature procedures.^36^ The ^1^H, ^13^C, and 2D NMR spectra were obtained using Bruker AV Neo 400 or Bruker Avance III 500 instruments (Bruker, Billerica, MA, USA). Chemical shifts in the ^1^H and ^13^C NMR spectra are given in parts per million (ppm) with reference to the residual proton signal of the deuterated solvent. High-resolution mass spectrometry (HRMS) measurements were performed on a Waters Synapt HDMS spectrometer (Waters Corporation, Milford, MA, USA). ^13^C CP/MAS NMR spectrum was acquired with a Bruker Avance III 400 spectrometer. The sample was spin at 8 kHz MAS frequency in a 4 mm ZrO_2_ rotor. Adamantane was used as a secondary chemical shift reference, with the selected resonance set at δ = 38.48 ppm. The relaxation delay in experiment was set to 1.3 × T1 time measured for phase with a saturation recovery experiment.

#### 2-([1,1′-Biphenyl]-4-yl)-4,4,5,5-tetramethyl-1,3,2-dioxaborolane (**2**)

A mixture of 4-bromo-1,1′-biphenyl (1 g, 4.3 mmol), bis­(pinacolato)­diboron (1.42 g, 5.58 mmol), Pd­(dppf)­Cl_2_ (0.09 g, 0.13 mmol), and potassium acetate (1.26 g, 12.9 mmol) in dry toluene (30 mL) was degassed and stirred at 85 °C for 24 h. After this time, water (ca. 30 mL) was added to the reaction mixture, and the mixture was extracted with dichloromethane (3 × 30 mL). The combined organic layers were dried over anhydrous MgSO_4_, filtered, and concentrated under reduced pressure. The crude product was purified by column chromatography on silica gel (petroleum ether: dichloromethane = 1:1, *R*_*f*_ = 0.44) to give **2** (1 g, 83%) as a white solid. ^1^H NMR (400 MHz, CDCl_3_): δ 7.92–7.86 (m, 2H), 7.64–7.59 (m, 4H), 7.49–7.41 (m, 2H), 7.40–7.32 (m, 1H), 1.36 (s, 12H). The ^13^C NMR spectrum is consistent with that described in the literature.^41^

#### 2″,5″-Bis­(methylsulfinyl)-1,1′:4′,1″:4″,1‴:4‴,1‴′-quinquephenyl (**3**)

A mixture of 1,4-dibromo-2,5-bis­(methylsulfinyl)­benzene (**1**) (0.23 g, 0.64 mmol), **2** (0.49 g, 1.70 mmol), and Pd­(PPh_3_)_4_ (0.016 g, 0.014 mmol) was placed in a three-necked flask, and 2 M K_2_CO_3_ solution (11.3 mL), tetrabutylammonium bromide (0.19 g), and dry toluene (37.5 mL) were added. The reaction mixture was degassed and stirred at 95 °C for 24 h. After cooling to room temperature, the reaction mixture was extracted with dichloromethane (3 × 30 mL). The combined organic layers were dried over anhydrous MgSO_4_, filtered, and concentrated under reduced pressure. The crude product was preliminarily purified by column chromatography on silica gel (chloroform, *R*_*f*_ = 0.22). The fraction containing the product was washed with methanol (20 mL). The precipitate was collected by suction filtration and dried under vacuum, yielding compound **3** as a mixture of diasteroisomers in 2:1 ratio (0.25 g, 77%) as a white solid. The diastereomers were not separated and were used as a mixture in the next step of the synthesis. ^1^H NMR (500 MHz, CDCl_3_): δ 8.16 (s, 2H), 8.15 (s, 2H), 7.76–7.71 (m, 8H), 7.70–7.66 (m, 8H), 7.58 (d, *J* = 8.2 Hz, 4H, minor isomer), 7.55 (d, *J* = 8.2 Hz, 4H, major isomer), 7.52–7.47 (m, 8H), 7.43–7.38 (m, 4H), 2.54 (s, 6H, C*H*_3_, major isomer), 2.47 (s, 6H, C*H*_3_, minor isomer). ^13^C NMR (126 MHz, CDCl_3_): δ 147.14 (minor isomer), 147.00 (major isomer), 141.82, 139.99, 139.41, 135.66 (major isomer), 135.48 (minor isomer), 129.57 (minor isomer), 129.51 (major isomer), 129.01, 127.93, 127.74, 127.16, 125.87 (minor isomer), 125.82 (major isomer), 41.64 (*C*H_3_, minor isomer), 41.48 (*C*H_3_, major isomer). HRMS (APCI^+^) *m*/*z*: [M + H]^+^ calcd for C_32_H_27_O_2_S_2_ 507.1452; found, 507.1454.

#### 2,8-Diphenylbenzo­[1,2-*b*:4,5-*b*′]­bis­[*b*]­benzothiophene (diPh-BBBT)

A mixture of **3** (0.15 g, 0.29 mmol), phosphorus pentoxide (0.17 g, 0.59 mmol), and trifluoromethanesulfonic acid (86 mL) was stirred in the dark at room temperature for 72 h. The reaction mixture was poured into ice–water (100 mL). The yellow precipitate was collected by suction filtration and dried under vacuum. The residue was assumed to be the corresponding sulfonium salt. Demethylation of the solid was achieved by refluxing in anhydrous pyridine (30 mL) for 12 h. After cooling the reaction mixture to room temperature, a large volume of water (150 mL) was added to precipitate the product, which was collected by suction filtration and dried under vacuum to give **diPh-BBBT** (0.12 g, 92%). ^1^H NMR (400 MHz, CDCl_3_): δ 8.63 (s, 2H), 8.28 (d, *J* = 8.2 Hz, 2H), 8.09 (s, 2H), 7.77–7.66 (m, 6H), 7.54–7.46 (m, 4H), 7.44–7.37 (m, 2H). Due to the very low solubility of **diPh-BBBT** in commonly used deuterated solvents, recording the ^13^C NMR spectra was not feasible. HRMS (APCI^+^) *m*/*z*: [M + H]^+^ calcd for C_30_H_19_S_2_ 443.0928; found, 443.0928. Elemental analysis for C_30_H_18_S_2_ (442.08): calcd C 81.41, H 4.10, S 14.49; found, C 81.09, H 4.17, S 14.32.

### Ultraviolet–Visible and Photoluminescence Spectroscopy

Electronic absorption spectra were obtained using a Shimadzu UV–VIS 2700 spectrophotometer (Shimadzu Scientific, Kyoto, Japan). Photoluminescence measurements were conducted with a FluoroMax + spectrofluorometer (Horiba Scientific, Kyoto, Japan). All experiments were carried out at room temperature in 1 cm cuvettes. For measurements in solution, spectrophotometric-grade DCM and DMSO were used. The photoluminescence spectra of **diPh-BBBT** were recorded upon excitation at their corresponding absorption maxima.

### Thermogravimetric Analysis

TGA was performed on a TGA 2950 instrument (TA Instruments, Eden Prairie, MN, USA). Measurements were carried out under a nitrogen atmosphere with samples heated from 20 to 800 °C at a heating rate of 10 °C/min. The decomposition temperature (*T*_d_) was defined as the point corresponding to a 5% loss in sample mass.

### Differential Scanning Calorimetry

Thermal behavior of the synthesized compound was further examined by differential scanning calorimetry using a DSC3 (Mettler Toledo), with heating conducted from 0 to 320 °C at a heating rate of 5 °C/min. Data were recorded under a nitrogen atmosphere with a flow rate of 40 mL/min.

### Quantum-Chemical Calculations

Quantum-chemical calculations were performed using Gaussian 09 (Revision D.01).^42^ Vertical electronic excitation energies and frontier orbital energies were calculated using TD-DFT with the PBE0 functional^43^ and the 6-311+G­(2d,p) basis set,^44^ incorporating solvent effects via the IEF-PCM implicit solvation model parametrized for DCM. These excited-state calculations were based on geometries optimized at the same level of theory, including the D3­(BJ) dispersion correction^45^ and the 6-311G­(2d,p) triple-ζ basis set.^44^ Internal reorganization energies, adiabatic ionization potentials (IPs), and adiabatic electron affinities (EAs) were evaluated in the gas phase using the M06-2X functional^46^ with the D3 dispersion correction^47^ and the def2-TZVPD basis set^48^ downloaded from the Basis Set Exchange (BSE).^49^ Adiabatic IPs were obtained from the energy difference between the optimized cationic states, and the optimized neutral species. Electron affinities were computed analogously as the difference between the energies of the optimized neutral molecule and its optimized anionic state. Hole (λ_h_) and electron (λ_e_) reorganization energies were derived using Nelsen’s four-point adiabatic potential method, as employed in previous studies of organic semiconductors and charge transport materials.^50,51^ For all geometry optimizations, the “ultrafine” integration grid and tight convergence criteria for geometry and energy were applied.

### X-ray Diffraction Analysis

X-ray diffraction measurements were performed using a Malvern Panalytical Empyrean diffractometer (Malvern Panalytical, Almelo, The Netherlands) equipped with a Cu Kα X-ray source (λ = 1.54178 Å). Data were collected in the 2θ range from 1° to 45° with a step size of 0.02° and a counting time of 1 s per step. The diffractometer was operated at 45 kV and 40 mA. XRD analysis was performed on a sample of mechanically oriented film. The resulting diffraction patterns were indexed using the EVA software suite, and crystallographic parameters were calculated from the observed diffraction maxima.

### Polarized Optical Microscopy

Polarized optical microscopy was performed using a Nikon Eclipse E400 Pol polarizing light microscope equipped with a SANYO VCC-3770P camera. The instrument was used to analyze films of **diPh-BBBT** deposited on glass substrates. The microscope was operated in transmission mode with crossed polarizers to visualize the birefringence and crystalline texture of the prepared films. Films were prepared by method mechanical orientation of the material on glass surfaces.

### Atomic Force Microscopy Analyses

Atomic force microscopy analyses were performed on a Nanosurf Flex Axiom system equipped with a C3000 controller (Nanosurf AG, Switzerland). Measurements were conducted in intermittent-contact (tapping) mode using standard silicon cantilevers with nominal spring constant ∼40 N/m and resonance frequency ∼300 kHz. Scan areas of 1 μm × 1 μm were recorded at 512 × 512 pixels, with drive amplitude set to 50% and a scan rate of 0.5–1 Hz to minimize tip–sample interaction forces and avoid sample damage. Topography and phase images were collected simultaneously in each measurement. Raw AFM data were processed using SPIP software (Image Metrology, Denmark) to extract quantitative information about surface roughness (*R*_q_ value), grain dimensions, and surface feature heights.

### Charge Transport Calculations

Charge-carrier mobility at 300 K was evaluated using the semiclassical Marcus hopping formalism combined with kinetic Monte Carlo simulations. To accurately represent the solid-state transport landscape, the intermolecular transfer integrals *J*_*ij*_ were explicitly calculated for molecular dimers constructed from the powder X-ray diffraction (XRD)-determined crystal structure. This approach ensured that the electronic coupling in the transport model corresponded to the experimentally observed molecular packing. Internal reorganization energy λ was obtained from DFT, while the relative positions, intermolecular separations, and mutual orientations of adjacent molecules were directly derived from the XRD-based packing motifs. The hopping rate between sites *i* and *j* was then evaluated using the Marcus expression

$$k_{ij}=\frac{2\pi }{\hbar }\frac{|J_{ij}{|}^{2}}{\sqrt{4\pi \lambda k_{B}T}}\exp\left[-\frac{{\left(\Delta E_{ij}+\lambda \right)}^{2}}{4\lambda k_{B}T}\right]$$

where *k*_B_ is the Boltzmann constant, *T* is the temperature, and Δ*E*_*ij*_ = *E*_*j*_ – *E*_*i*_ is the site-energy difference associated with a given hopping event. Under an applied electric field, the driving term was written as

$$\Delta E_{ij}=E_{j}-E_{i}-F\Delta x_{ij}$$

where *F* denotes the field strength along the transport direction and Δ*x*_*ij*_ is the projection of the intermolecular displacement onto the field axis. The dimer-derived transfer integrals *J*_*ij*_ were used as direct input parameters for all transport channels in the kinetic Monte Carlo procedure, ensuring that the calculated macroscopic mobility explicitly incorporated both the intrinsic molecular electronic structure and the experimentally resolved crystal packing. Such a protocol provides a physically consistent bridge between DFT-level molecular parameters, XRD-resolved intermolecular geometry, and mesoscopic charge-transport simulations at the device-relevant scale.

## Supplementary Material



## Notes

Supporting Information includes the NMR spectra of compound **3** (^1^H, ^13^C, COSY, HMBC, and HSQC) and **diPh-BBBT** (^1^H and ^13^C CP/MAS). Due to the very low solubility of **diPh-BBBT** in commonly used deuterated solvents (such as DMSO-*d*_6_, DMF-*d*_6_, CDCl_3_, Acetone-*d*_6_, C_6_D_6_), even at elevated temperatures, recording the ^13^C NMR spectrum in solution was not feasible. Supporting Information also includes the HRMS spectrum of **diPh-BBBT**, TGA and DSC analyses, and the atomic coordinates of **diPh-BTBT** and **diPh-BBBT** (PDF)
