# Supplementary material for: 2,8‑Diphenylbenzo[1,2‑b:4,5‑b′]bis[b]benzothiophene: A New Thienoacene Derivative for Potential Organic Electronic Applications
Source: ACS Omega. 2026 Jul 13;11(29):44234–42. doi: 10.1021/acsomega.6c04431 (PMC13425487; doi:10.1021/acsomega.6c04431)
Supplement: Supplementary file 1 [file ao6c04431_si_001.pdf]

Supporting Information

2,8-Diphenylbenzo[1,2-b:4,5-b']bis[b]benzothiophene: A New Thienoacene Derivative for Potential Organic Electronic Applications

Aneta Rzewnicka *[a], Remigiusz Żurawiński,[a] Jerzy Krysiak,[a] and Tomasz Makowski *[b]

[a] *Division of Organic Chemistry, Centre of Molecular and Macromolecular Studies, Polish Academy of Sciences, Sienkiewicza 112, 90-363 Lodz, Poland; aneta.rzewnicka@cbmm.lodz.pl (A. R.); remigiusz.zurawinski@cbmm.lodz.pl (R. Ż.); jerzy.krysiak@cbmm.lodz.pl (J. K.)*

[b] *Department of Polymeric Nano-materials, Centre of Molecular and Macromolecular Studies Polish Academy of Science, Sienkiewicza 112, 90-363 Lodz, Poland; tomasz.makowski@cbmm.lodz.pl (T. M.)*

*Correspondence: aneta.rzewnicka@cbmm.lodz.pl (A. R.), tomasz.makowski@cbmm.lodz.pl (T. M.)

Table of Contents:

1. NMR spectra……………………………………………………………S2-S6
2. HRMS spectra……………………………………………………………S6
3. TGA and DSC analysis……………………………………………………S7
4. Theoretical calculations …………………………………………………S7-S14

1. NMR Spectra

1.1 NMR Spectra of 2'',5''-bis(methylsulfinyl)-1,1':4',1'':4'',1''':4''',1''''-quinquephenyl (**3**)

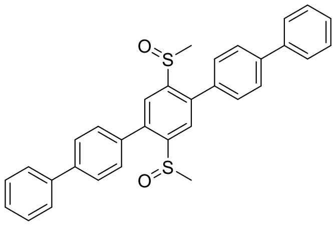

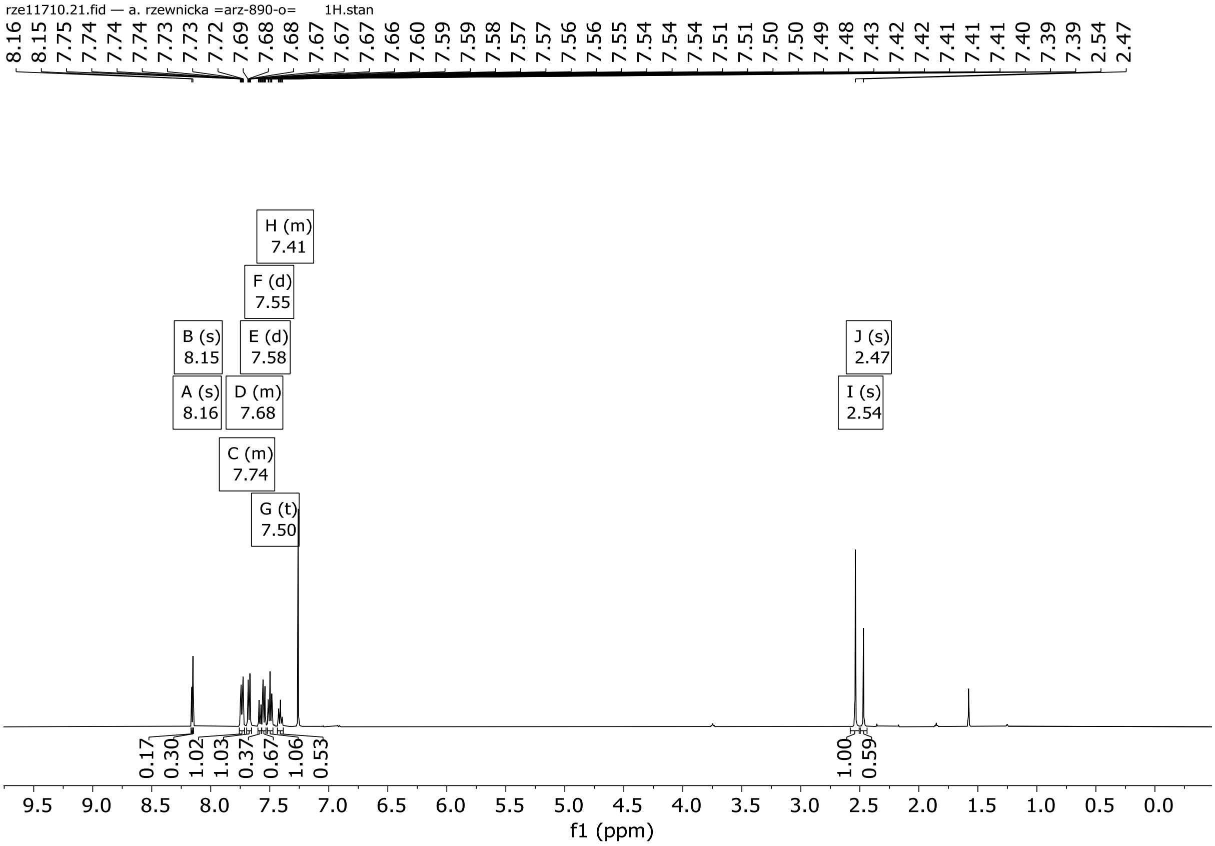

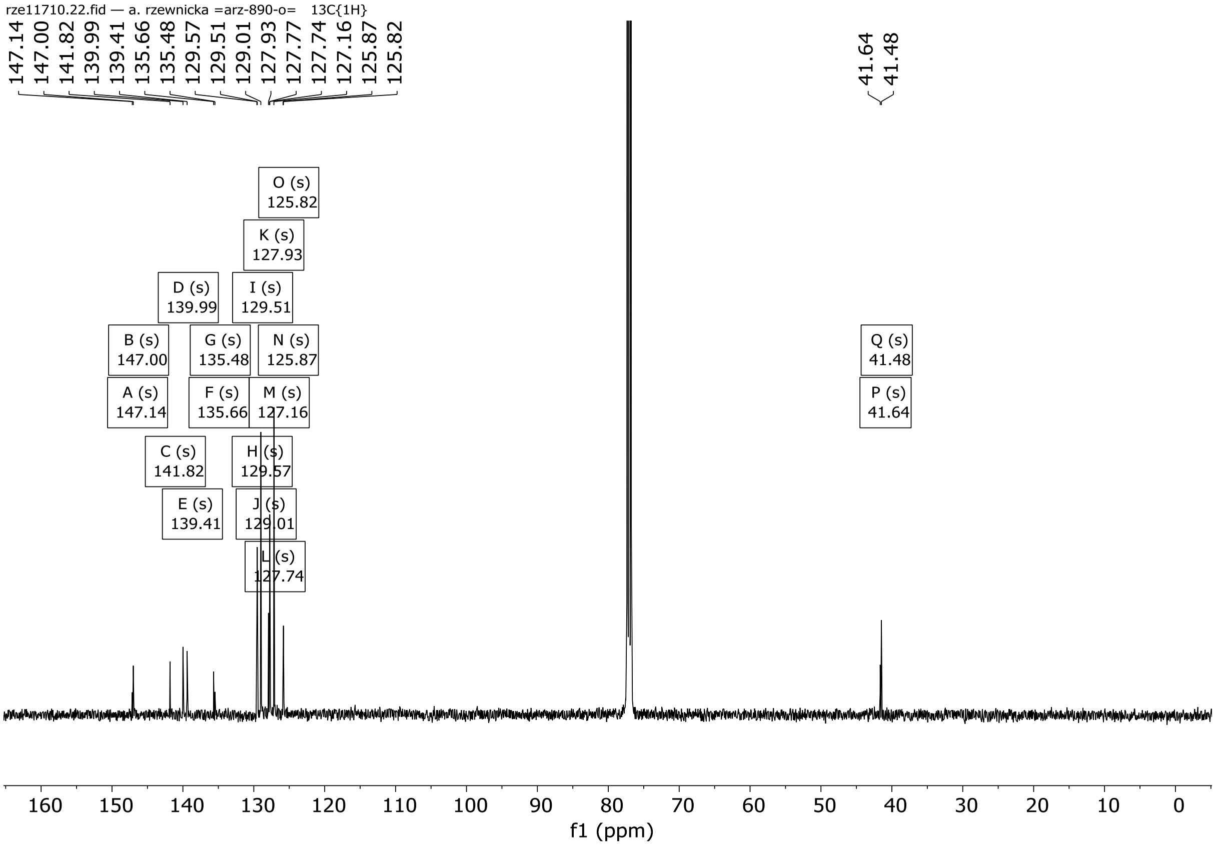
rze11710.22.fid — a. rzewnicka =arz-890-o= 13C{1H}
147.14
147.00
141.82
139.99
139.41
135.66
135.48
129.57
129.51
129.01
127.93
127.77
127.74
127.16
125.87
125.82
41.64
41.48
O (s) 125.82
K (s) 127.93
D (s) 139.99
I (s) 129.51
B (s) 147.00
G (s) 135.48
N (s) 125.87
Q (s) 41.48
A (s) 147.14
F (s) 135.66
M (s) 127.16
P (s) 41.64
C (s) 141.82
H (s) 129.57
E (s) 139.41
J (s) 129.01
L (s) 127.74
160 150 140 130 120 110 100 90 80 70 60 50 40 30 20 10 0
f1 (ppm)

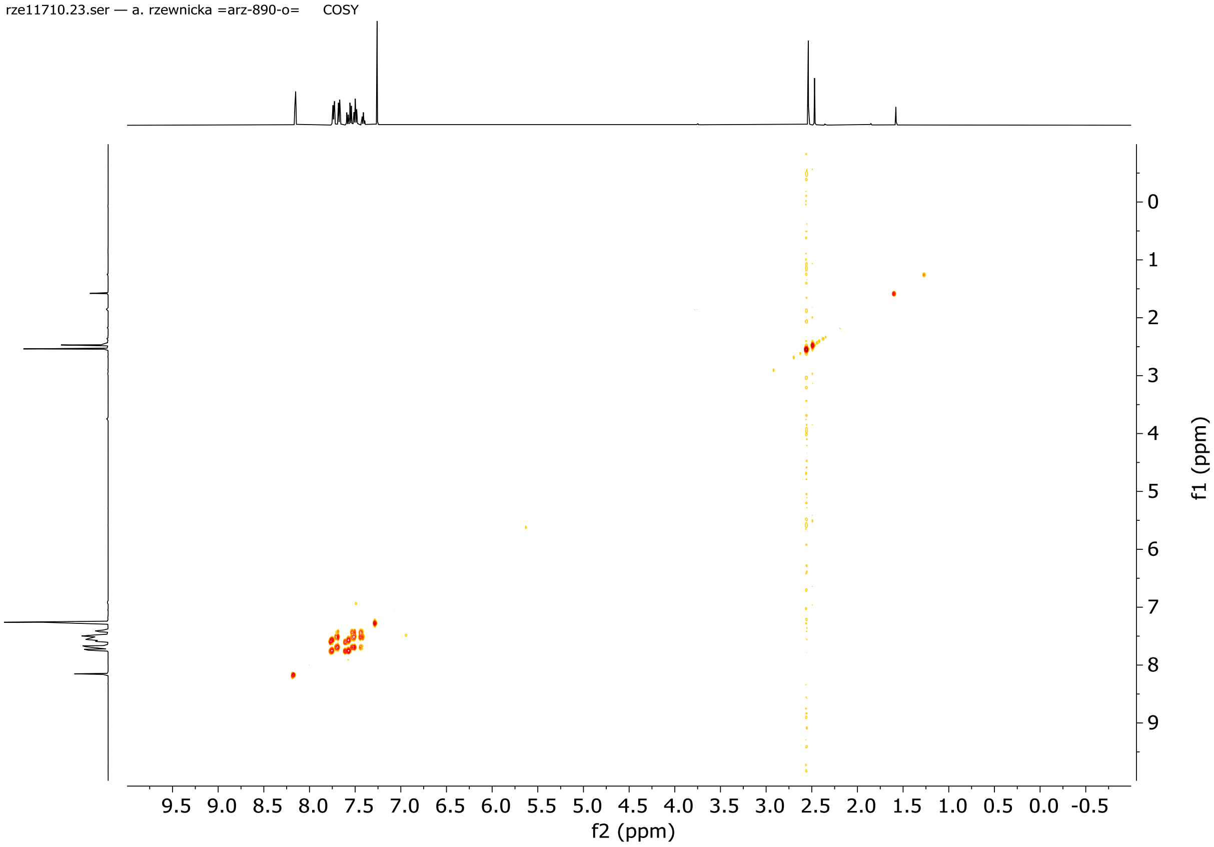
rze11710.23.ser — a. rzewnicka =arz-890-o= COSY
9.5 9.0 8.5 8.0 7.5 7.0 6.5 6.0 5.5 5.0 4.5 4.0 3.5 3.0 2.5 2.0 1.5 1.0 0.5 0.0 -0.5
f2 (ppm)
0 1 2 3 4 5 6 7 8 9
f1 (ppm)

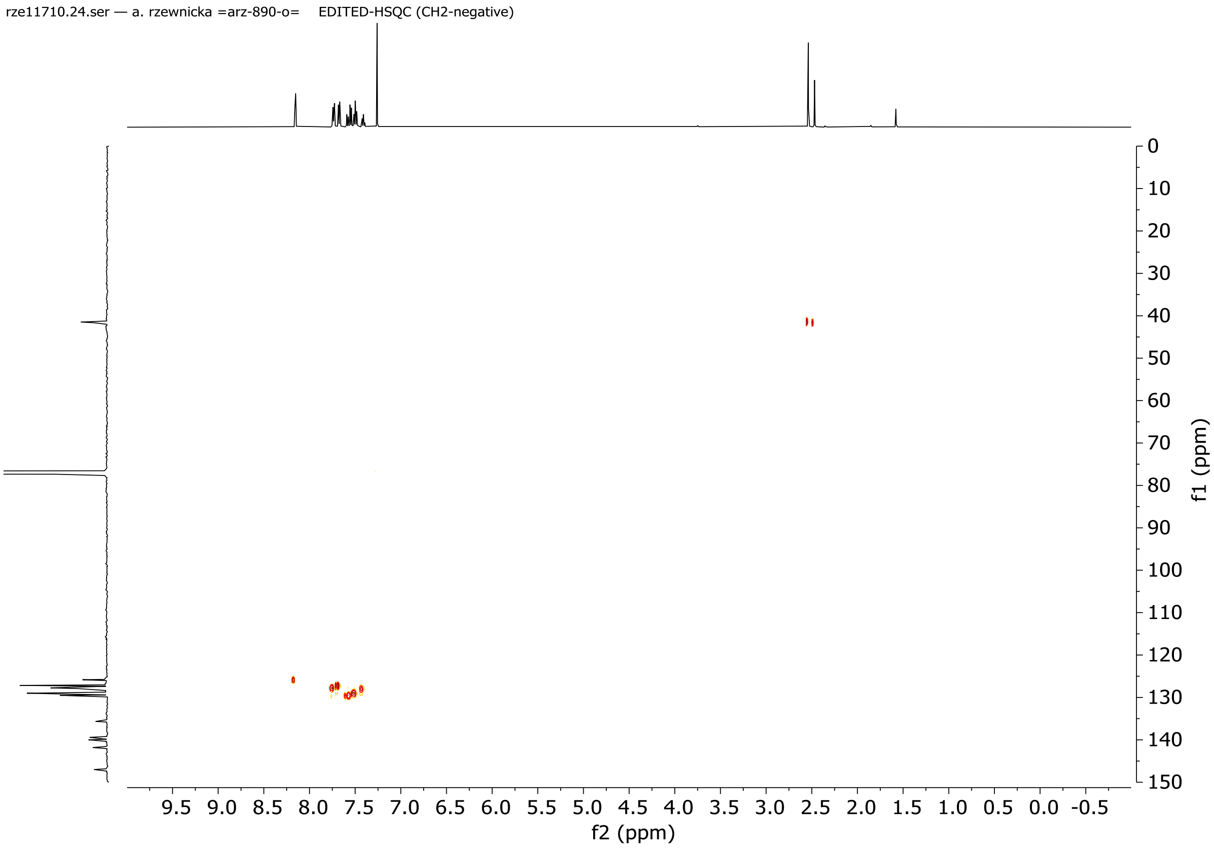
rze11710.24.ser — a. rzewnicka =arz-890-o= EDITED-HSQC (CH2-negative)
f1 (ppm)
f2 (ppm)

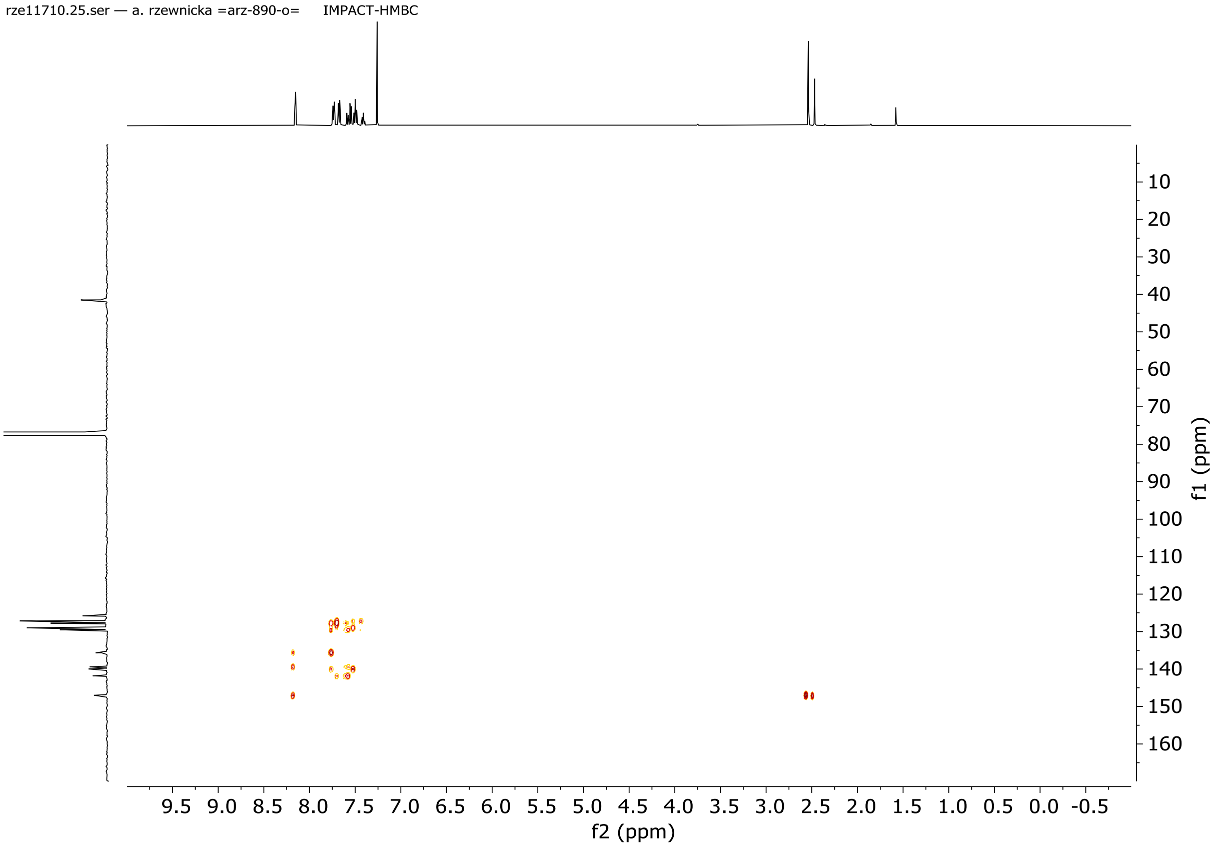
rze11710.25.ser — a. rzewnicka =arz-890-o= IMPACT-HMBC
f1 (ppm)
f2 (ppm)

1.2 NMR Spectra of 2,8-diphenylbenzo[1,2-*b*:4,5-*b*']bis[*b*]benzothiophene (**diPh-BBBT**)

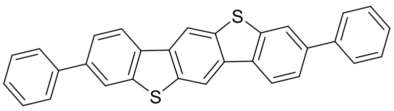

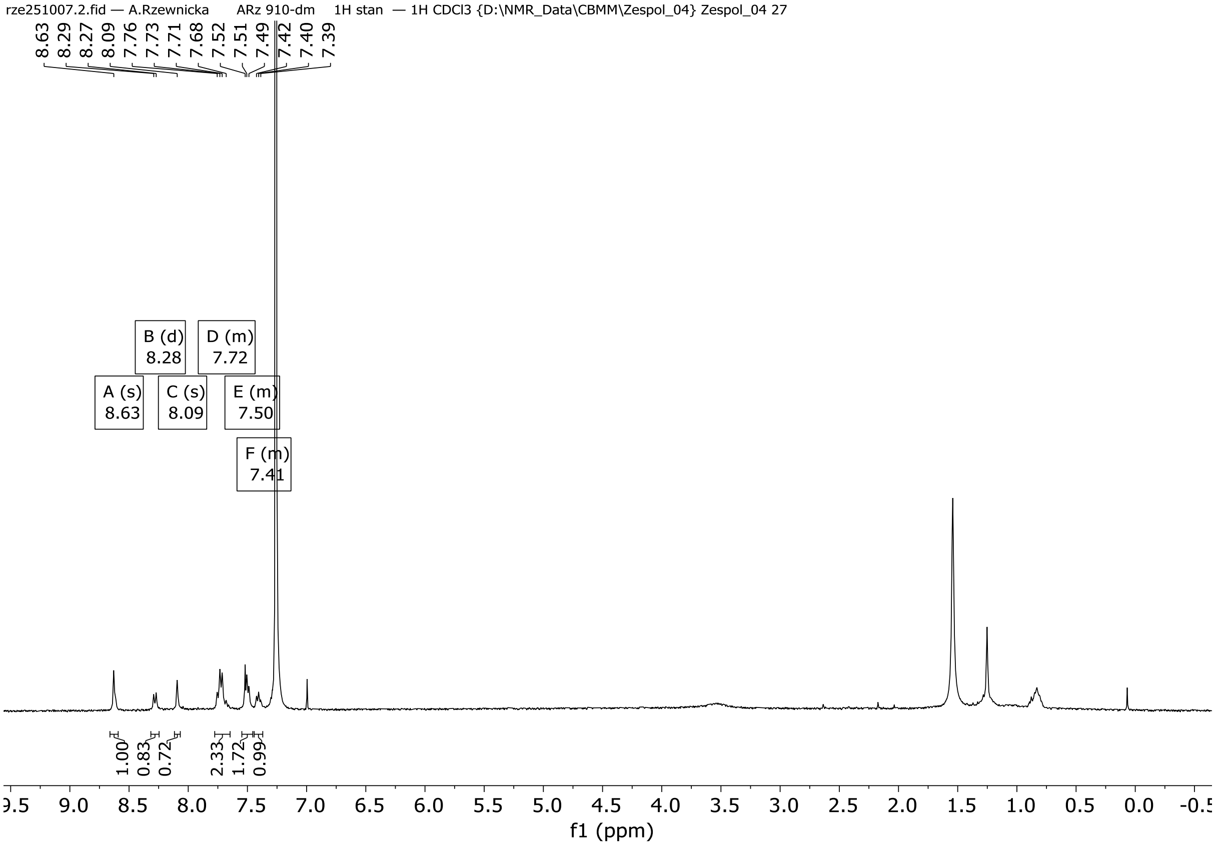

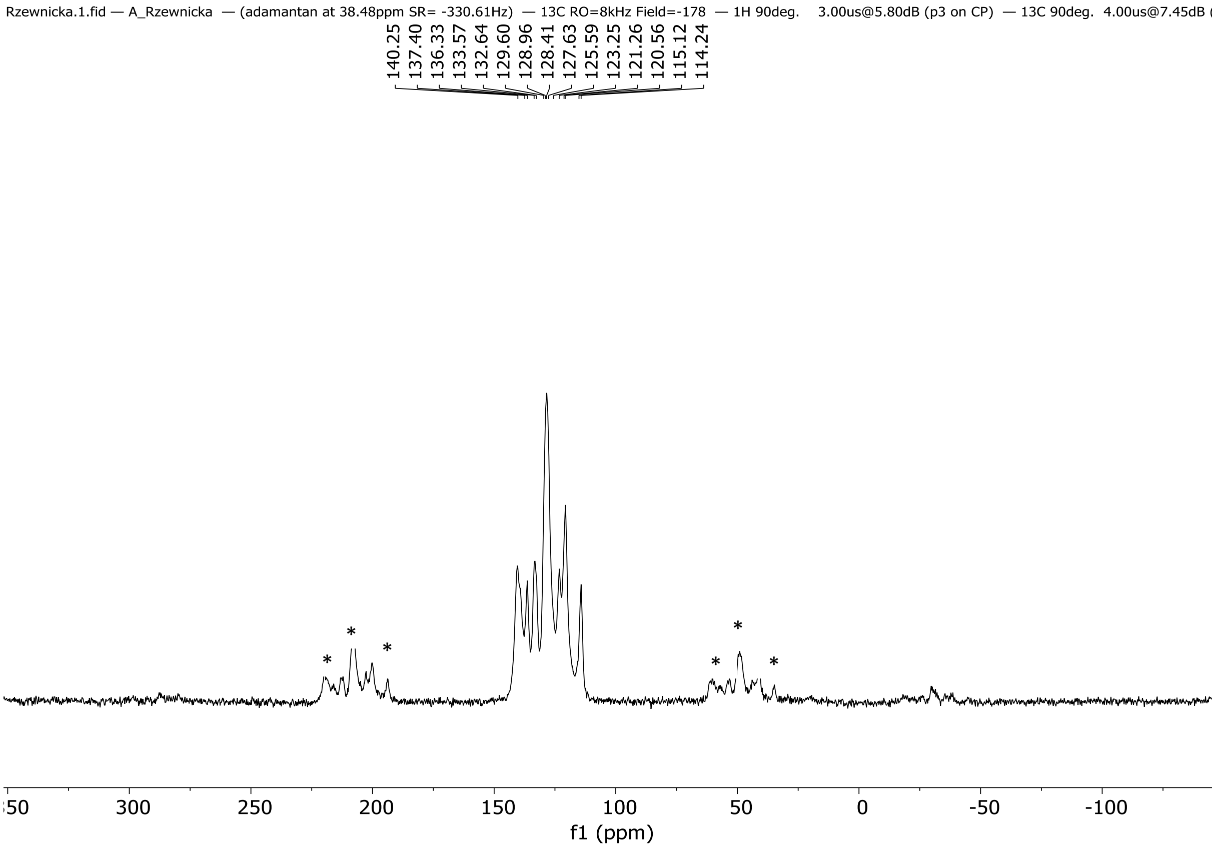

* - side bands

2. HRMS spectrum of 2,8-diphenylbenzo[1,2-*b*:4,5-*b*']bis[*b*]benzothiophene (**diPh-BBBT**)

Elemental Composition Report **Page 1**

Single Mass Analysis
Tolerance = 5.0 PPM / DBE: min = -1.5, max = 60.0
Element prediction: Off
Number of isotope peaks used for i-FIT = 9

Monoisotopic Mass, Even Electron Ions
68 formula(e) evaluated with 1 results within limits (all results (up to 1000) for each mass)
Elements Used:
C: 0-50 H: 0-100 O: 0-3 S: 0-2
250307_ARz_871k_1_3_apci_A 27 (0.294) Cm (27:33-7:12)

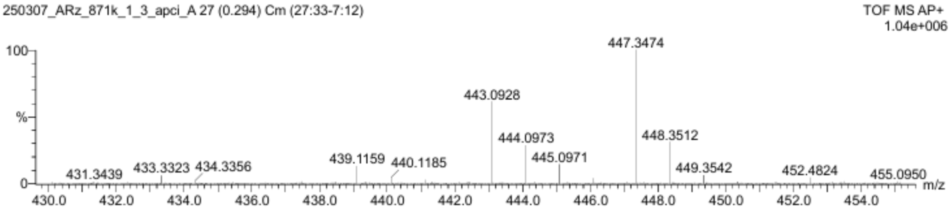

Minimum:				-1.5				
Maximum:		5.0	5.0	60.0				
Mass	Calc. Mass	mDa	PPM	DBE	i-FIT	Norm	Conf(%)	Formula
443.0928	443.0928	0.0	0.0	21.5	2073.3	n/a	n/a	C30 H19 S2

3. TGA and DSC analysis

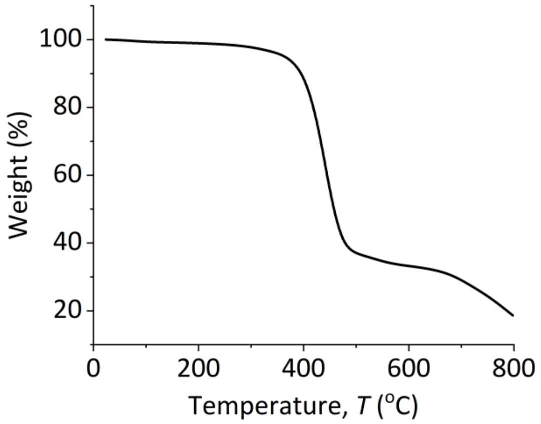

Figure S1. TGA analysis for **diPh-BBBT**.

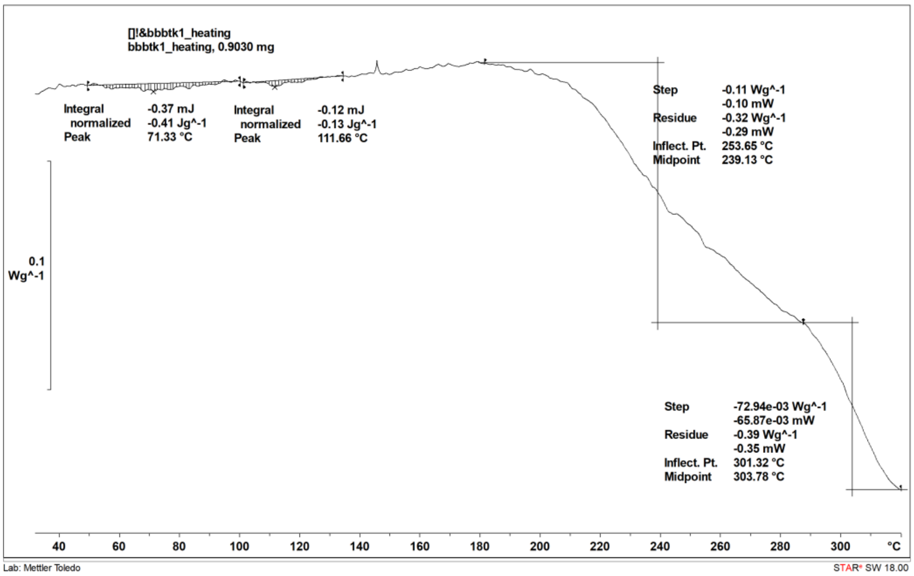

Figure S2. DSC analysis for **diPh-BBBT**.

4. Theoretical calculations

Atomic coordinates of **diPh-BTBT** and **diPh-BBBT** optimized at the TDDFT PBE0/6-311+G(2d,p)//D3(BJ)- PBE0/6-311G(2d,p) level in DCM (IEFPCM solvation model):

diPh-BTBT

0 1

C	-2.22482048	0.59578258	0.07181409
C	-1.67711200	-0.69019198	-0.12420953
C	-0.25317415	-0.62942557	-0.10250283
C	0.25317415	0.62942557	0.10250283
C	2.22482048	-0.59578258	-0.07181409
C	1.67711200	0.69019198	0.12420953
C	3.90759681	1.55767380	0.27224536
H	4.57759639	2.39475624	0.43080090
C	2.54690602	1.76959307	0.29799020
H	2.15177731	2.76634228	0.46030077
C	3.59418278	-0.80514536	-0.09644806
H	3.99467331	-1.79688837	-0.27276812

C	-3.59418278	0.80514536	0.09644806
H	-3.99467331	1.79688837	0.27276812
C	-3.90759681	-1.55767380	-0.27224536
H	-4.57759639	-2.39475624	-0.43080090
C	-2.54690602	-1.76959307	-0.29799020
H	-2.15177731	-2.76634228	-0.46030077
S	-0.99497595	1.82494324	0.28118070
S	0.99497595	-1.82494324	-0.28118070
C	4.45257047	0.27564190	0.07672035
C	-4.45257047	-0.27564190	-0.07672035
C	-5.91441334	-0.07903226	-0.05504367
C	-6.75566928	-1.03501675	0.51810634
C	-6.49127750	1.06735675	-0.60561041
C	-8.12878819	-0.84936169	0.54210694
H	-6.32648033	-1.92189657	0.97159553
C	-7.86433223	1.25296354	-0.58192554
H	-5.85789954	1.80940004	-1.07974630
C	-8.68930004	0.29547427	-0.00764752
H	-8.76392572	-1.59987300	0.99985256
H	-8.29291802	2.14648293	-1.02252995
H	-9.76369861	0.44035027	0.01047557
C	5.91441334	0.07903226	0.05504367
C	6.75566928	1.03501675	-0.51810634
C	6.49127750	-1.06735675	0.60561041
C	8.12878819	0.84936169	-0.54210694
H	6.32648033	1.92189657	-0.97159553
C	7.86433223	-1.25296354	0.58192554
H	5.85789954	-1.80940004	1.07974630
C	8.68930004	-0.29547427	0.00764752
H	8.76392572	1.59987300	-0.99985256
H	8.29291802	-2.14648293	1.02252995
H	9.76369861	-0.44035027	-0.01047557

diPh-BBBT

0 1

C	3.42581290	-0.78478203	-0.11616170
C	2.79204100	0.45324612	0.07533328
C	3.58585072	1.58675692	0.25080061
C	4.96012095	1.47321043	0.23159129
C	5.59046421	0.23251412	0.03997871
C	4.80608282	-0.90367939	-0.13573706
C	0.93297457	-0.99328925	-0.15627045
C	1.35302353	0.33923178	0.05540914
H	3.12537358	2.55571635	0.41024057
H	5.56924229	2.35515227	0.39235027
H	5.27277952	-1.86632291	-0.30978348
C	-1.35302353	-0.33923178	-0.05540914
C	-0.93297457	0.99328925	0.15627045
C	0.40265913	1.34290048	0.21289352
C	-0.40265913	-1.34290048	-0.21289352
C	-2.79204100	-0.45324612	-0.07533328
C	-3.42581290	0.78478203	0.11616170
H	0.70535929	2.37082736	0.37580075
H	-0.70535929	-2.37082736	-0.37580075
C	-3.58585072	-1.58675692	-0.25080061
H	-3.12537358	-2.55571635	-0.41024057
C	-4.80608282	0.90367939	0.13573706
H	-5.27277952	1.86632291	0.30978348

C	-5.59046421	-0.23251412	-0.03997871
C	-4.96012095	-1.47321043	-0.23159129
H	-5.56924229	-2.35515227	-0.39235027
S	-2.28414151	2.09392518	0.32924241
S	2.28414151	-2.09392518	-0.32924241
C	7.06235099	0.13343518	0.02537385
C	7.84078204	1.14218770	-0.54582701
C	7.71098063	-0.97109471	0.58137436
C	9.22330799	1.04816668	-0.56267463
H	7.35559758	1.99760179	-1.00320186
C	9.09348832	-1.06472839	0.56551424
H	7.12559899	-1.75296863	1.05317047
C	9.85574285	-0.05559488	-0.00698489
H	9.80955229	1.83817505	-1.01916256
H	9.57838782	-1.92679528	1.01034841
H	10.93748841	-0.12866954	-0.01937407
C	-7.06235099	-0.13343518	-0.02537385
C	-7.84078204	-1.14218770	0.54582701
C	-7.71098063	0.97109471	-0.58137436
C	-9.22330799	-1.04816668	0.56267463
H	-7.35559758	-1.99760179	1.00320186
C	-9.09348832	1.06472839	-0.56551424
H	-7.12559899	1.75296863	-1.05317047
C	-9.85574285	0.05559488	0.00698489
H	-9.80955229	-1.83817505	1.01916256
H	-9.57838782	1.92679528	-1.01034841
H	-10.93748841	0.12866954	0.01937407

Atomic coordinates of **diPh-BTBT** and **diPh-BBBT** optimized at the D3-M06-2X/def2-TZVPD level:

diPh-BTBT (neutral)

0 1

C	-2.22228221	0.59719374	0.08151953
C	-1.67965617	-0.68429464	-0.13666236
C	-0.24963544	-0.62644599	-0.11352840
C	0.24963544	0.62644599	0.11352840
C	2.22228221	-0.59719374	-0.08151953
C	1.67965617	0.68429464	0.13666236
C	3.90783972	1.55270212	0.30151472
H	4.57976610	2.38334601	0.47463868
C	2.54692036	1.76272679	0.33039789
H	2.14720711	2.75305126	0.51006737
C	3.59579773	-0.80418902	-0.10850119
H	3.99850223	-1.79050880	-0.30215429
C	-3.59579773	0.80418902	0.10850119
H	-3.99850223	1.79050880	0.30215429
C	-3.90783972	-1.55270212	-0.30151472
H	-4.57976610	-2.38334601	-0.47463868
C	-2.54692036	-1.76272679	-0.33039789
H	-2.14720711	-2.75305126	-0.51006737
S	-0.99409532	1.81551204	0.31241120
S	0.99409532	-1.81551204	-0.31241120
C	4.45083017	0.27293739	0.08341509
C	-4.45083017	-0.27293739	-0.08341509
C	-5.91831957	-0.07728075	-0.05794364
C	-6.75280632	-1.02218493	0.54025194
C	-6.49826651	1.05605513	-0.62917958
C	-8.12667651	-0.83798683	0.56913554

H	-6.31726550	-1.89625861	1.00840830
C	-7.87212204	1.24055738	-0.60132118
H	-5.86718612	1.78577128	-1.12142247
C	-8.69177076	0.29427303	-0.00159407
H	-8.75723295	-1.57730634	1.04596902
H	-8.30452388	2.12176209	-1.05748528
H	-9.76409507	0.43780398	0.02010496
C	5.91831957	0.07728075	0.05794364
C	6.75280632	1.02218493	-0.54025194
C	6.49826651	-1.05605513	0.62917958
C	8.12667651	0.83798683	-0.56913554
H	6.31726550	1.89625861	-1.00840830
C	7.87212204	-1.24055738	0.60132118
H	5.86718612	-1.78577128	1.12142247
C	8.69177076	-0.29427303	0.00159407
H	8.75723295	1.57730634	-1.04596902
H	8.30452388	-2.12176209	1.05748528
H	9.76409507	-0.43780398	-0.02010496

diPh-BTBT (cation)

1 2

C	-2.20637007	0.59710625	0.04834089
C	-1.66121628	-0.70228463	-0.10494981
C	-0.25746238	-0.65553896	-0.08579120
C	0.25746238	0.65553896	0.08579120
C	2.20637007	-0.59710625	-0.04834089
C	1.66121628	0.70228463	0.10494981
C	3.88861011	1.58423263	0.22190639
H	4.56071911	2.41986599	0.35680736
C	2.53552852	1.79738155	0.24369831
H	2.13612631	2.79500250	0.37640051
C	3.56177387	-0.81151999	-0.06298587
H	3.96440661	-1.80467403	-0.20997735
C	-3.56177387	0.81151999	0.06298587
H	-3.96440661	1.80467403	0.20997735
C	-3.88861011	-1.58423263	-0.22190639
H	-4.56071911	-2.41986599	-0.35680736
C	-2.53552852	-1.79738155	-0.24369831
H	-2.13612631	-2.79500250	-0.37640051
S	-0.97101565	1.83809003	0.22306913
S	0.97101565	-1.83809003	-0.22306913
C	4.43137625	0.28738575	0.07110136
C	-4.43137625	-0.28738575	-0.07110136
C	-5.88286612	-0.08404543	-0.05221609
C	-6.73168643	-1.06593347	0.47288805
C	-6.44562052	1.09403541	-0.55817145
C	-8.10051774	-0.86954706	0.49935656
H	-6.31501502	-1.96992883	0.89768687
C	-7.81609937	1.27956556	-0.54642011
H	-5.81069116	1.85084250	-1.00074163
C	-8.64645393	0.30083289	-0.01373343
H	-8.74420429	-1.62761860	0.92468516
H	-8.23980746	2.18560546	-0.95810587
H	-9.71794160	0.44981973	0.00107083
C	5.88286612	0.08404543	0.05221609
C	6.73168643	1.06593347	-0.47288805
C	6.44562052	-1.09403541	0.55817145
C	8.10051774	0.86954706	-0.49935656

H	6.31501502	1.96992883	-0.89768687
C	7.81609937	-1.27956556	0.54642011
H	5.81069116	-1.85084250	1.00074163
C	8.64645393	-0.30083289	0.01373343
H	8.74420429	1.62761860	-0.92468516
H	8.23980746	-2.18560546	0.95810587
H	9.71794160	-0.44981973	-0.00107083

diPh-BTBT (anion)

-1 2

C	-2.23612139	0.60394101	0.02001004
C	-1.66558439	-0.70302246	-0.06383897
C	-0.26872354	-0.64248825	-0.04815119
C	0.26872354	0.64248825	0.04815119
C	2.23612139	-0.60394101	-0.02001004
C	1.66558439	0.70302246	0.06383897
C	3.91750688	1.57750324	0.12656315
H	4.57743711	2.43134868	0.22015817
C	2.56127142	1.79635033	0.14252355
H	2.17097788	2.80320510	0.22897836
C	3.59293291	-0.81135240	-0.02755733
H	3.97877592	-1.81836393	-0.12641689
C	-3.59293291	0.81135240	0.02755733
H	-3.97877592	1.81836393	0.12641689
C	-3.91750688	-1.57750324	-0.12656315
H	-4.57743711	-2.43134868	-0.22015817
C	-2.56127142	-1.79635033	-0.14252355
H	-2.17097788	-2.80320510	-0.22897836
S	-0.99326653	1.84611469	0.12154232
S	0.99326653	-1.84611469	-0.12154232
C	4.48220410	0.28563376	0.04288502
C	-4.48220410	-0.28563376	-0.04288502
C	-5.92971270	-0.08805249	-0.04182144
C	-6.81390396	-1.08709475	0.40566060
C	-6.51127294	1.11405703	-0.48532005
C	-8.18476355	-0.89866122	0.40254923
H	-6.41192854	-2.01499147	0.79140943
C	-7.88262774	1.30281820	-0.48063646
H	-5.87386704	1.89989086	-0.86944058
C	-8.73806668	0.29925029	-0.03907587
H	-8.82999525	-1.69120009	0.76326961
H	-8.28999553	2.24111063	-0.83866837
H	-9.81015963	0.44712726	-0.03741545
C	5.92971270	0.08805249	0.04182144
C	6.81390396	1.08709475	-0.40566060
C	6.51127294	-1.11405703	0.48532005
C	8.18476355	0.89866122	-0.40254923
H	6.41192854	2.01499147	-0.79140943
C	7.88262774	-1.30281820	0.48063646
H	5.87386704	-1.89989086	0.86944058
C	8.73806668	-0.29925029	0.03907587
H	8.82999525	1.69120009	-0.76326961
H	8.28999553	-2.24111063	0.83866837
H	9.81015963	-0.44712726	0.03741545

diPh-BBBT (neutral)

0 1

C	3.42430475	-0.78417129	-0.12800015

C	2.79555788	0.45058371	0.08251384
C	3.58749439	1.58238736	0.27559251
C	4.96219225	1.47013044	0.25443185
C	5.59002795	0.23148273	0.04316695
C	4.80787852	-0.90109607	-0.14911056
C	0.93559385	-0.99189512	-0.17164614
C	1.35097102	0.33694182	0.06072484
H	3.12501692	2.54593626	0.45044125
H	5.57369569	2.34598249	0.42842405
H	5.27584821	-1.85892778	-0.33837666
C	-1.35097102	-0.33694182	-0.06072484
C	-0.93559385	0.99189512	0.17164614
C	0.40243445	1.33861510	0.23369710
C	-0.40243445	-1.33861510	-0.23369710
C	-2.79555788	-0.45058371	-0.08251384
C	-3.42430475	0.78417129	0.12800015
H	0.70575173	2.36247766	0.41238889
H	-0.70575173	-2.36247766	-0.41238889
C	-3.58749439	-1.58238736	-0.27559251
H	-3.12501692	-2.54593626	-0.45044125
C	-4.80787852	0.90109607	0.14911056
H	-5.27584821	1.85892778	0.33837666
C	-5.59002795	-0.23148273	-0.04316695
C	-4.96219225	-1.47013044	-0.25443185
H	-5.57369569	-2.34598249	-0.42842405
S	-2.28476192	2.08543948	0.36027372
S	2.28476192	-2.08543948	-0.36027372
C	7.06738971	0.13287744	0.02613487
C	7.84133503	1.12880344	-0.57041176
C	7.71680804	-0.95793077	0.60508828
C	9.22454012	1.03555555	-0.58982233
H	7.35204680	1.97064296	-1.04447944
C	9.10002688	-1.05115142	0.58726145
H	7.13190155	-1.72639405	1.09533232
C	9.85928998	-0.05478364	-0.01074959
H	9.80804779	1.81318016	-1.06560907
H	9.58651980	-1.90041461	1.04946715
H	10.93887745	-0.12721730	-0.02495404
C	-7.06738971	-0.13287744	-0.02613487
C	-7.84133503	-1.12880344	0.57041176
C	-7.71680804	0.95793077	-0.60508828
C	-9.22454012	-1.03555555	0.58982233
H	-7.35204680	-1.97064296	1.04447944
C	-9.10002688	1.05115142	-0.58726145
H	-7.13190155	1.72639405	-1.09533232
C	-9.85928998	0.05478364	0.01074959
H	-9.80804779	-1.81318016	1.06560907
H	-9.58651980	1.90041461	-1.04946715
H	-10.93887745	0.12721730	0.02495404

diPh-BBBT (cation)

1 2

C	3.43141642	-0.73195675	-0.11280064
C	2.80549706	0.50359679	0.08979655
C	3.60341272	1.62522426	0.27367201
C	4.98266123	1.49134438	0.25217977
C	5.60459678	0.25269031	0.04771821
C	4.80643129	-0.87929291	-0.13853405

C	0.95035936	-0.96858346	-0.16190806
C	1.35781742	0.38429331	0.06738715
H	3.15869292	2.59779797	0.44264869
H	5.60064050	2.36331537	0.42054483
H	5.26246183	-1.84336421	-0.32296821
C	-1.35781742	-0.38429331	-0.06738715
C	-0.95035936	0.96858346	0.16190806
C	0.40087094	1.35357284	0.22929956
C	-0.40087094	-1.35357284	-0.22929956
C	-2.80549706	-0.50359679	-0.08979655
C	-3.43141642	0.73195675	0.11280064
H	0.66953796	2.38747464	0.40461485
H	-0.66953796	-2.38747464	-0.40461485
C	-3.60341272	-1.62522426	-0.27367201
H	-3.15869292	-2.59779797	-0.44264869
C	-4.80643129	0.87929291	0.13853405
H	-5.26246183	1.84336421	0.32296821
C	-5.60459678	-0.25269031	-0.04771821
C	-4.98266123	-1.49134438	-0.25217977
H	-5.60064050	-2.36331537	-0.42054483
S	-2.27013622	2.02103962	0.33677650
S	2.27013622	-2.02103962	-0.33677650
C	7.07768242	0.13890798	0.02645756
C	7.85576871	1.13617726	-0.56213255
C	7.71158760	-0.96689321	0.59395583
C	9.23694536	1.02717099	-0.58557709
H	7.37695448	1.98731565	-1.03037368
C	9.09321875	-1.07102974	0.57612346
H	7.12311932	-1.73499014	1.08096044
C	9.85875445	-0.07533730	-0.01525491
H	9.82868136	1.80116880	-1.05586553
H	9.57342102	-1.92698080	1.03130280
H	10.93727790	-0.15821086	-0.03137370
C	-7.07768242	-0.13890798	-0.02645756
C	-7.85576871	-1.13617726	0.56213255
C	-7.71158760	0.96689321	-0.59395583
C	-9.23694536	-1.02717099	0.58557709
H	-7.37695448	-1.98731565	1.03037368
C	-9.09321875	1.07102974	-0.57612346
H	-7.12311932	1.73499014	-1.08096044
C	-9.85875445	0.07533730	0.01525491
H	-9.82868136	-1.80116880	1.05586553
H	-9.57342102	1.92698080	-1.03130280
H	-10.93727790	0.15821086	0.03137370

diPh-BBBT (anion)

-1 2

C	3.44292950	-0.79913591	-0.07057491
C	2.78528613	0.45120403	0.05010004
C	3.59902112	1.59773035	0.16115345
C	4.97115235	1.48093332	0.14259578
C	5.61880662	0.23600130	0.02333582
C	4.81345215	-0.91514761	-0.08171058
C	0.93049198	-1.00614314	-0.09997125
C	1.37102278	0.33909168	0.03684126
H	3.14124654	2.57291674	0.27451982
H	5.57119041	2.37505969	0.26088012
H	5.27069732	-1.88875213	-0.20823644

C	-1.37102278	-0.33909168	-0.03684126
C	-0.93049198	1.00614314	0.09997125
C	0.39542695	1.36150265	0.13823436
C	-0.39542695	-1.36150265	-0.13823436
C	-2.78528613	-0.45120403	-0.05010004
C	-3.44292950	0.79913591	0.07057491
H	0.69428161	2.39677369	0.24410078
H	-0.69428161	-2.39677369	-0.24410078
C	-3.59902112	-1.59773035	-0.16115345
H	-3.14124654	-2.57291674	-0.27451982
C	-4.81345215	0.91514761	0.08171058
H	-5.27069732	1.88875213	0.20823644
C	-5.61880662	-0.23600130	-0.02333582
C	-4.97115235	-1.48093332	-0.14259578
H	-5.57119041	-2.37505969	-0.26088012
S	-2.29049079	2.11095307	0.21003961
S	2.29049079	-2.11095307	-0.21003961
C	7.08169406	0.13778335	0.01927888
C	7.88750040	1.18503979	-0.45546188
C	7.74050983	-1.01011507	0.48777284
C	9.26900863	1.09189600	-0.45562199
H	7.41669310	2.07287808	-0.85772766
C	9.12219444	-1.10502385	0.48087235
H	7.15568489	-1.82692033	0.89103752
C	9.90218678	-0.05472209	0.01107460
H	9.85806837	1.91741318	-0.83714396
H	9.59568001	-2.00362327	0.85809064
H	10.98196338	-0.12853869	0.00743902
C	-7.08169406	-0.13778335	-0.01927888
C	-7.88750040	-1.18503979	0.45546188
C	-7.74050983	1.01011507	-0.48777284
C	-9.26900863	-1.09189600	0.45562199
H	-7.41669310	-2.07287808	0.85772766
C	-9.12219444	1.10502385	-0.48087235
H	-7.15568489	1.82692033	-0.89103752
C	-9.90218678	0.05472209	-0.01107460
H	-9.85806837	-1.91741318	0.83714396
H	-9.59568001	2.00362327	-0.85809064
H	-10.98196338	0.12853869	-0.00743902
